# A Network Pharmacology Approach to Evaluating the Efficacy of Chinese Medicine Using Genome-Wide Transcriptional Expression Data

**DOI:** 10.1155/2013/915343

**Published:** 2013-05-12

**Authors:** Leihong Wu, Yi Wang, Jing Nie, Xiaohui Fan, Yiyu Cheng

**Affiliations:** ^1^Pharmaceutical Informatics Institute, College of Pharmaceutical Sciences, Zhejiang University, Hangzhou 310058, China; ^2^Chiatai Qingchunbao Pharmaceutical Co., Ltd., Hangzhou 310023, China

## Abstract

The research of multicomponent drugs, such as in Chinese Medicine, on both mechanism dissection and drug discovery is challenging, especially the approaches to systematically evaluating the efficacy at a molecular level. Here, we presented a network pharmacology-based approach to evaluating the efficacy of multicomponent drugs by genome-wide transcriptional expression data and applied it to Shenmai injection (SHENMAI), a widely used Chinese Medicine composed of red ginseng (RG) and Radix Ophiopogonis (RO) in clinically treating myocardial ischemia (MI) diseases. The disease network, MI network in this case, was constructed by combining the protein-protein interactions (PPI) involved in the MI enriched pathways. The therapeutic efficacy of SHENMAI, RG, and RO was therefore evaluated by a network parameter, namely, network recovery index (NRI), which quantitatively evaluates the overall recovery rate in MI network. The NRI of SHENMAI, RG, and RO were 0.876, 0.494, and 0.269 respectively, which indicated SHENMAI exerts protective effects and the synergistic effect of RG and RO on treating myocardial ischemia disease. The successful application of SHENMAI implied that the proposed network pharmacology-based approach could help researchers to better evaluate a multicomponent drug on a systematic and molecular level.

## 1. Background

Chinese Medicine, featured as having “multiple ingredients and multiple targets,” has been widely used to treat complex diseases for decades [1]. So far, the therapeutic efficacy of Chinese Medicine has been conventionally evaluated by a few pharmacological biomarkers or pathological endpoint indicators, such as tumor size, myocardial infarct size, or serum enzymes indicators. For instance, LV function was widely applied as the golden indicator to evaluate the drug's effect on the heart by echocardiography [2]. However, phenotype indicators can barely reflect the systematical impacts caused by multiple components [3, 4], which often involve multiple cross-talk pathways and mechanisms. Therefore, how to evaluate the efficacy of Chinese Medicine on a systematic and molecular level is challenging, especially when treating complex diseases such as cancer [5] and cardiovascular disease [6].

It would be promising to reveal a drug's efficacy via high throughput technology [7], such as microarray and genome-wide association study (GWAS). Previous studies demonstrated that microarray could be used to find potential disease biomarkers [8–13], which was valuable in prognosis prediction and mechanism explanation. However, a conventional microarray analytical approach to finding outcome-related genes also has its limitations [14, 15]. For instance, due to the large number of features and relatively small number of samples in omics data, statistically significant DEGs might in fact not have valuable biological meanings; meanwhile, moderately expressed biomarkers would be overlooked if a high cutoff was set to filter out noise. In addition, most approaches to finding significant biomarkers did not take the multiplex interactions into consideration.

Recently, network based analyses such as network pharmacology emerged and have become a powerful tool to systematically reveal complex biological relationships [16, 17]. Unlike strategies to find solely expressed genes, network based studies consider connection relationships between genes to find biologically important nodes. For instance, previous studies indicated that hub gene nodes in disease-gene and PPI networks play key roles in biological systems [18, 19]. The combination of a network based analysis and microarray studies would be valuable in disease and drug study [20–22] to discover potential biomarkers [23] and for disease classification [24].

**Figure 1:**
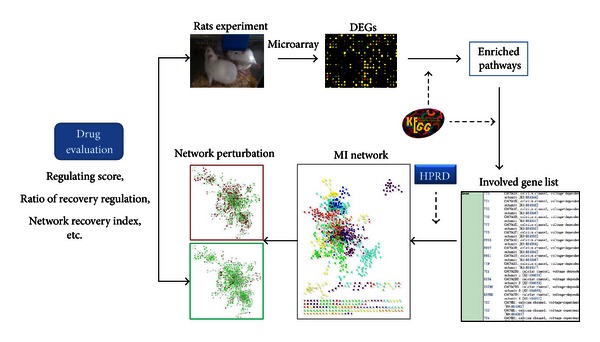
Study workflow. Nodes in the MI network were selected based on enriched MI-related pathways according to microarray results, and connections between nodes in the MI network were collected from the PPI database. The network perturbations caused by MI and SHENMAI were analyzed via network annotated analysis and were finally quantitatively evaluated by an indicator named network recovery index (NRI).

In this study, for the first time, network pharmacology is introduced and applied to the evaluation of the efficacy of Shenmai injection (SHENMAI) in treating myocardial ischemia (MI). SHENMAI, composed of red ginseng (RG, *Panax ginseng* C. A. Mey, steamed and dry) and Radix Ophiopogonis (RO, *Ophiopogon japonicus *(L. f.) Ker-Gawl, root), is a Chinese Medicine that is widely used in clinically treating ischemic heart disease [25–27]. As shown in Figure 1,a PPI network of myocardial ischemia (MI network) was first constructed by combining the protein-protein interactions (PPI) involved in MI enriched pathways. The expression data was then imported and revealed the recovery rate of SHENMAI in the MI network. The drug efficacy based on network analysis was evaluated by a network parameter called network recovery index (NRI). The result of this study quantitatively showed that SHENMAI exerts protective effects on treating myocardial ischemia, as it made the biological network recover from disease state to normal state. In addition, through the comparison of NRI we also showed the synergistic effect of RG and RO on treating myocardial ischemia,on a systematic and molecular level.

## 2. Materials and Methods

### 2.1. Quality Control of SHENMAI

**Figure 2:**
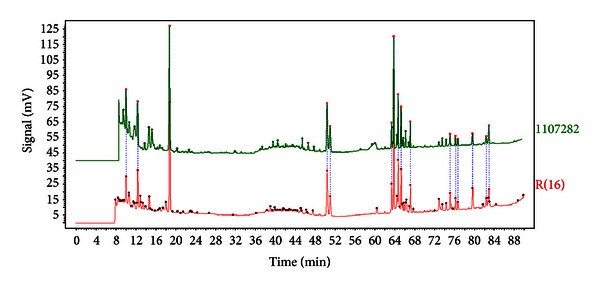
HPLC fingerprints of SHENMAI. The similarity between the fingerprint of batch no. 1107282 (upper) and the reference fingerprint (bottom) is 0.99, which is significantly over 0.85, that is, the threshold required by the State Food and Drug Administration of China.

SHENMAI (no. 1107282) extracts of RG and RO, and the vehicle in the form of an injection, were supplied by *Chiatai Qingchunbao* Pharmaceutical Co., Ltd. (Hangzhou, China). The HPLC fingerprinting assay was performed to estimate the batch-to-batch consistency of SHENMAI [28]. As revealed in Figure 2, the HPLC fingerprints of SHENMAI and the similarity between the fingerprint of batch no. 1107282 and the reference fingerprint is 0.99, which is significantly over 0.85, that is, the threshold required by the State Food and Drug Administration of China [28]. The result of the HPLC fingerprinting assay suggested that the batch-to-batch consistency of SHENMAI is high enough for further experiments.

### 2.2. Rat Experiment

Male Sprague-Dawley rats (180–220 g) were purchased from Shanghai SLAC Laboratory Animal Co., Ltd. A rat model of myocardial infarction was induced by permanent occlusion of the left anterior descending coronary artery. Briefly, after anesthetized using diethyl ether, the rats were fixed with the hair over the heart picked, then underwent a left thoracotomy and were ligated on the left anterior descending coronary artery. The control (sham) group was given the same surgery but without ligation. SHENMAI and the vehicle were given via intraperitoneal injection for 7 days in rats with myocardial ischemia. In detail, 10 mL/kg of SHENMAI and the corresponding dose for RG and RO were given to the rats, respectively. The dosage of SHENMAI and its components used was calculated in accordance with clinical consumption and was confirmed by our preliminary study. On the eighth day, the rats were anaesthetized by intraperitoneal injection of 0.2 mL/100 g anesthetic, which was prepared by mixing diazepam and ketamine in the ratio of equality, for measuring echocardiography using a VisualSonics Vevo 770TM in vivo microimaging system equipped with an RMV-707B cardiovascular scanhead (Toronto, ON, canada). Then the border between the infarct and noninfarct left ventricle area in the rat heart was harvested to extract mRNA for genome-wide transcriptomic analysis.

### 2.3. RNA Extraction and Purification

Total RNA was extracted using TRIZOL Reagent (Life technologies, Carlsbad, CA, US) following the manufacturer's instructions and checked for a RIN number to inspect RNA integrity by an Agilent Bioanalyzer 2100 (Agilent technologies, Santa Clara, CA, US). Qualified total RNA was further purified by RNeasy mini kit (QIAGEN, GmBH, Germany) and RNase-Free DNase Set (QIAGEN, GmBH, Germany). 

### 2.4. Genome-Wide Transcriptomic Experiment

Total RNA was amplified, labeled, and purified by using GeneChip 3'IVT Express Kit (Affymetrix, Santa Clara, CA, US) following the manufacturer's instructions to obtain biotin-labeled cRNA. Array hybridization and wash were performed using GeneChip Hybridization, Wash and Stain Kit (Affymetrix, Santa Clara, CA, US) in Hybridization Oven 645 (Affymetrix, Santa Clara, CA, US) and Fluidics Station 450 (Affymetrix, Santa Clara, CA, US) following the manufacturer's instructions. Slides were scanned by GeneChip Scanner 3000 (Affymetrix, Santa Clara, CA, US) and Command Console Software 3.1 (Affymetrix, Santa Clara, CA, US) with default settings. Raw data were stored on ArrayTrack [29], a java-based microarray analysis tool developed by the US FDA and normalized by MAS 5.0 algorithm.

### 2.5. MI Network Construction

MI network was constructed based on the MI enriched pathways and PPI knowledge. To define MI enriched pathways, a widely used approach of Welch *t*-test with a threshold of *P* value <0.01 and fold change >1.5 was applied to find out the significant genes according to the microarray expression profiles. In this study, the significant genes differentially expressed between Myocardial ischemia (MI) group and control group were used for further pathway enrichment analysis.

Pathway enrichment analysis was then applied to find the significant enriched pathways of these DEGs. ArrayTrack [29] was used to find significant pathways with the KEGG database [30]. 

In this study, the nodes of the MI network are the genes involved in the MI enriched pathways, and the edges are the protein-protein interactions between these genes. Since protein-protein interactions (PPIs) for rats were relatively few in number that could hardly reflect the overall network relationships between genes (statistics from BioGRID [31], only 2089 interactions in total compared to 106344 in human), we used human PPI data, which was imported from the Human Protein Reference Database (HPRD) [32] to build the MI network. Homologous genes in humans were found by ArrayTrack. Only gene-gene interactions whose source and target genes were both located in the same enriched pathway were used. In other words, two genes were linked in the MI network only when they were located in the same pathway and showed a PPI interaction with each other.

### 2.6. Network Recovery Index Calculation

The network recovery index (NRI) was used as a quantitative index to evaluate the network perturbation recovery from MI to normal state by treatment. To calculate the NRI, the regulating score (RS) was first calculated to evaluate the influence of MI and SHENMAI on each node in the network, which was calculated by the average ratio of expression data between MI/treatment and control samples formula ((a)). In formula ((a)), EV_disease_ refers to the expression value of the MI group, EV_normal_ represents the expression value of the normal group, and EV_drug_ is the expression value of the drug group. In this study, nodes with RS_disease_ > 0.5 were considered as upregulating nodes and nodes with RS_disease_ < −0.33 were defined as downregulating nodes, because these nodes represented an absolute fold-change > 1.5 between MI and control samples in microarray:

$$\begin{array}{cc} \left(\text{a}\right) & \text{RS}=\{\begin{array}{cc} \frac{\left({\text{EV}}_{\text{disease}}-{\text{EV}}_{\text{normal}}\right)}{{\text{EV}}_{\text{normal}}}, & \text{for}\;\text{disease},\\ \frac{\left({\text{EV}}_{\text{drug}}-{\text{EV}}_{\text{normal}}\right)}{{\text{EV}}_{\text{normal}}}, & \text{for}\;\text{treatment}. \end{array} \end{array}$$

The ratio of recovery regulation (Rr) was measured as the ratio of nodes with a positive regulating level, which was determined by the difference ratio of the regulating score between the drug and the disease (formula ((b)). Regulating level = 1 indicated that the regulation trend of the drug was contrary to that of the disease, which meant a recovery regulation; and regulating level = 0 was considered as the opposite. For instance, the Rr score of upregulating nodes was calculated as the ratio of upregulating nodes with RL = 1 in the MI network

$$\begin{array}{cc} \left(\text{b}\right) & \text{Rr}=\sum_{i\in M}\frac{{\text{RL}}_{i}}{M},\;\text{where}\;{\text{RL}}_{i}=\{\begin{array}{cc} 1, & \frac{\left({\text{RS}}_{i,\text{disease}}-{\text{RS}}_{i,\text{drug}}\right)}{{\text{RS}}_{i,\text{disease}}}>0\\ 0, & \frac{\left({\text{RS}}_{i,\text{disease}}-{\text{RS}}_{i,\text{drug}}\right)}{{\text{RS}}_{i,\text{disease}}}<0. \end{array} \end{array}$$

NRI was finally calculated as an average Rr score combining up, downregulating nodes and all the nodes in the MI network, as was described in formula ((c)), where Rr_up_ and Rr_down_ refer to the Rr score of upregulating nodes and downregulating nodes and Rr_all_ refers to the ratio of recovery regulation of all nodes:

$$\begin{array}{cc} \left(\text{c}\right) & \text{NRI}=\frac{\left({\text{Rr}}_{\text{up}}+{\text{Rr}}_{\text{down}}+{\text{Rr}}_{\text{all}}\right)}{3}. \end{array}$$

## 3. Results and Discussion

### 3.1. MI Network Construction

A combined algorithm with simple *t*-test and fold change was used to find DEGs. By a threshold of *P* < 0.01 and absolute fold change >1.5, 1957 significant expressed probes were selected by comparison between MI and control samples, involving 1376 unique genes. Pathway enrichment analysis was then applied to these genes using the KEGG pathway database, and 27 significant enriched pathways were found by ArrayTrack (*P* < 0.05). These 27 pathways involved 10 metabolism pathways, 10 cellular process pathways, 4 human disease pathways, 2 environmental information processing pathways, and DNA replication. For instance, The adipocytokine signaling pathway (KEGG id: rno04920) showed a *P*-value = 0.026, and dilated cardiomyopathy (KEGG id: rno05414) showed a *P*-value <1.0*e* − 8. A detailed list of the 27 significant pathways used in this study is in Supplement Table S1 (see Table S1 in supplementary materials available online at http://dx.doi.org/10.1155/2013/915343).

According to the KEGG database, a total of 1478 genes were involved in these 27 significant pathways. The Orthologene database in ArrayTrack was used to convert these genes from rat to human. As a result, 905 genes have been found to have at least one interaction with other genes (including self-loops), and all the 2618 interactions were involved. 

**Figure 3:**
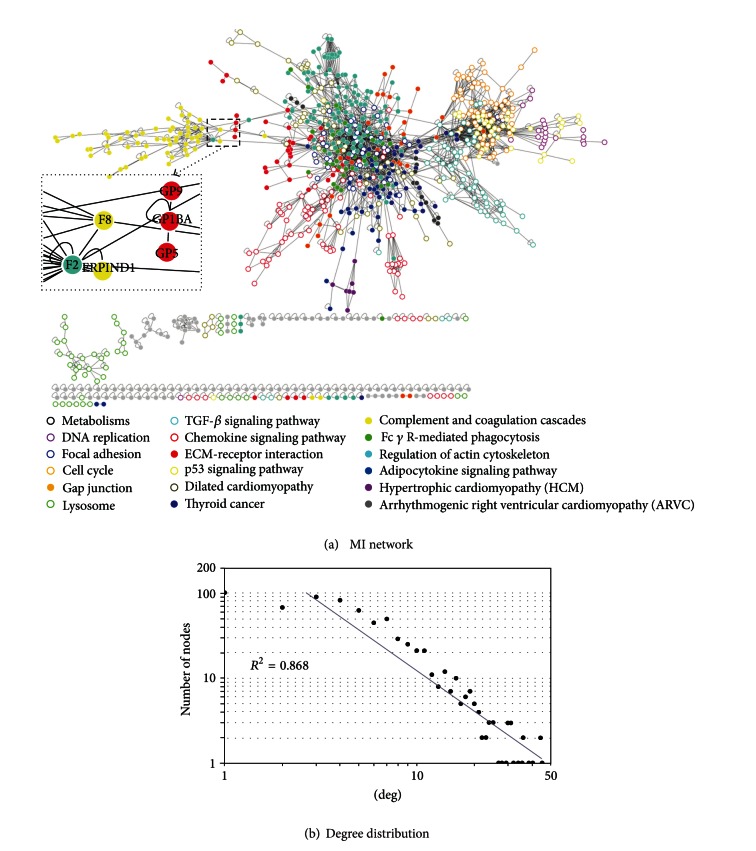
(a) MI network which was visualized by Cytoscape. Genes in different pathways were differently colored and shaped. (b) The degree of nodes in the MI network followed a power-law distribution, which that the indicated MI network is scale-free.

The MI network was constructed based on these genes and interactions, and was visualized by Cytoscape (version 2.8.0) [33] (Figure 3(a)). In this network, 814 out of 905 genes were connected to other nodes, and 700 of them formed a giant component with 2370 links, including 149 genes with node degree > 10. As most biological networks are scale-free, which means the nodes degrees follow a power law rather than Poisson distribution [34], we also tested whether the MI network was scale-free, just like other biological networks. As a result, the node degree distribution of the giant component suits well a power law degree (*R* ^2^ = 0.868), indicating that MI the network was scale-free like Figure 3(b).

### 3.2. Recovery Regulation by SHENMAI

The number of up-and downregulating nodes in the giant component by MI was 177 and 106; the number of up-and downregulating nodes by SHENMAI was 112 and 60, respectively. The result showed that the number of regulated nodes by SHENMAI was smaller than in MI. 

**Figure 4:**
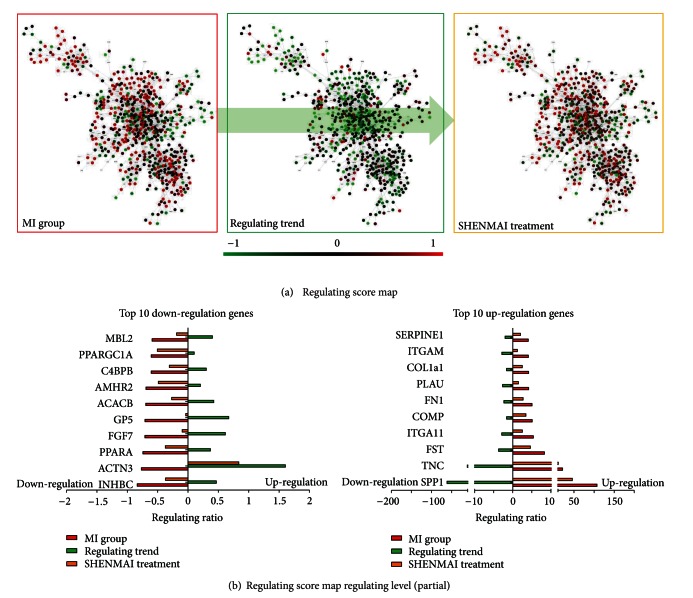
(a) The regulating score map of MI and SHENMAI and the regulating trend map of SHENMAI. The regulating trends of the original MI network (disease state) and the SHENMAI network were similar but contrary to the regulating trend map of SHENMAI, which indicated that SHENMAI alleviated the disease status but did not severely reverse the biological system. (b) The top 10 up and down regulated genes in the original disease network. As shown, the directions (up or down) of the regulating score (MI and SHENMAI) and regulating trend are different, which means that the SHENMAI treatment more or less recovers these top regulated genes, indicating that SHENMAI could help the biological system recover from the disease state.

Regulating score maps of SHENMAI and MI were drawn based on the regulating score of the MI group and SHENMAI treatment (Figure 4(a), left and right), and the regulating trend map shows the difference between SHENMAI and MI regulating score (Figure 4(a), middle). In general, we found the regulating score maps were quite similar, and the regulating trend map was quite different compared to the regulating score maps. As presented, most nodes with an upregulation the MI regulating score map were downregulated in the regulating trend map, and vice versa. This result implied that SHENMAI might have a reverse effect on MI. To validate this surmise, we calculated the regulating level of SHENMAI. As a result, 77.9% nodes (489/628) had a regulating level = 1, which confirmed that SHENMAI tries to relieve MI or to help the status recover from MI. In addition, the Rr scores were even more significant among up- and downregulating nodes. In fact, 90.96% (161/177) upregulated genes and 90.57% (96/106) downregulated genes were recovered (partial top results shown in Figure 4(b)). 

### 3.3. Network Recovery Index

The NRI was used to quantitatively evaluate the efficacy of the drug, based on the ability of the drug to recover the network from MI to normal. The NRI was a value between 0 and 1, and a higher NRI represented a good efficacy in treating MI in our study. As mentioned above, the ratio of recovery regulation of SHENMAI, Rr_up_, Rr_down_, and Rr_all_, were 0.9096, 0.9057, and 0.7787, respectively. So, the NRI of SHENMAI was 0.865. 

We also calculated the NRI of RG, and RO, which were solely used to treat MI. The NRI of SHENMAI, RG and RO were 0.865, 0.425, and 0.271, respectively (Table 1). Both RG and RO had significantly lower NRIs than SHENMAI, and the ratio of recovery regulation of up-and downregulating nodes of RG and RO did not show a better performance trend than all the other nodes. The result of the NRI indicated that RG and RO did not have the same drug efficacy as that of SHENMAI when treating MI (*P* = 0.0002 and 0.0006).

### 3.4. Efficacy Validation with Echocardiography Experiments

**Figure 5:**
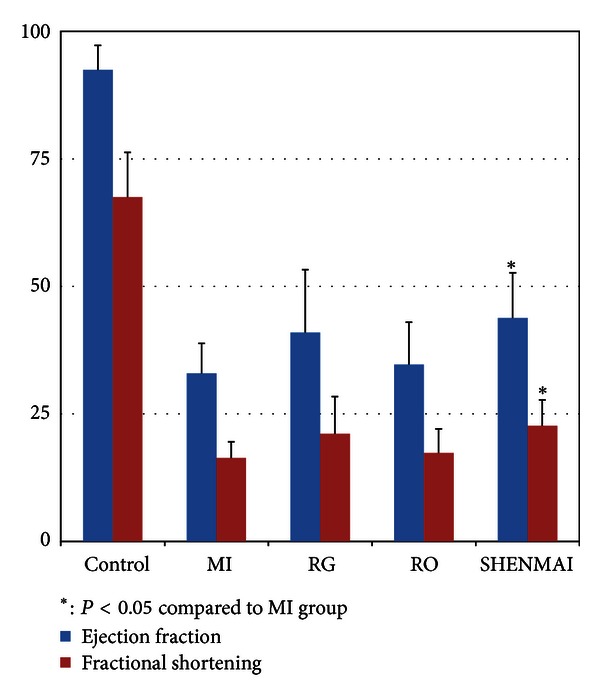
Echocardiography results of Control, MI, SHENMAI-treated, RG-treated, and RO-treated rats at one week after MI injury. SHENMAI exhibits a significantly greater impairment in percentage ejection fraction (EF) and fractional shortening (FS) related to MI controls; the error bar was drawn with mean ± SD.

In our study, LV function was used as the golden indicator to evaluate the drug efficacy of SHENMAI, RG, and RO in the development of cardiac failure after myocardial ischemia by using echocardiography after 7 days of treatment. As shown in Figure 5,the rats in the model group showed severely decreased cardiac contractility and fractional shorting after myocardial ischemic injury. By contrast, SHENMAI-treated rats exhibited a significantly greater impairment in percentage ejection fraction (EF) and fractional shortening (FS) (*P* < 0.05) related to MI controls. However, individual treatment of RG and RO did not show significance to impair the cardiac function. 

The overall trends in EF and FS indicators of SHENMAI, RG, and RO were consistent with the NRI in the network based analysis, which showed SHENMAI had the best and significant efficacy performance according to both the echocardiography study and network based analysis, while RG and RO showed insignificant efficacy according to the echocardiography result. The accordance of the echocardiography result and NRI indicated that network pharmacology based analysis could reveal drug effects on treating MI on a molecula level related to phenotype indicators.

## 4. Conclusion

In this study, we presented a network-based approach to evaluating the efficacy of Chinese Medicine using genome-wide transcriptional expression data. By constructing a specified biological network of MI and analyzing the network regulation, we quantitatively evaluated the efficacy of the multicomponent drug SHENMAI in treating MI. The result indicates that SHENMAI has a significant efficacy in treating MI, which was represented by its ability to repair the MI network. Furthermore, comparative analysis of SHENMAI and its components RG and RO indicated that RG and RO have the synergistic effect on treating MI. This result was further validated by LV function via echocardiography experiments. Based on our study, we believe that evaluating the efficacy of Chinese Medicine on a systematic and molecular level will be a trend in future drug research.

## Supplementary Material

Supplement Table: Detailed information of 27 enriched pathways. KEGG pathway analysis tools embedded in ArrayTrack were used for pathway analysis, and pathway with Fisher p-value <0.05 was identified as the enriched pathway. 27 enriched pathways were eventually identified and their detailed information was listed in Supplement Table .

## Figures and Tables

**Table 1:** Regulating level and NRI of SHENMAI.

	SHENMAI	RG	RO	Num of nodes
Up	161 (91.0%)	87 (49.2%)	29 (16.4%)	177
Down	61 (93.8%)	32 (49.2%)	23 (35.4%)	65
ALL	489 (77.9%)	313 (49.8%)	182 (29.0%)	628
NRI	0.876	0.494	0.269	

## Abbreviations

DEG: Differentially expressed genes
EF: Ejection fraction
FS: Fractional shortening
HPRD: Human Protein Reference Database
LV function: Left ventricular function
MI: Myocardial ischemia
NRI: Network recovery index
PPI: Protein-protein interaction
RG: Red ginseng, *Panax ginseng*, C.A. Mey steamed and dry
RO: Radix Ophiopogonis, *Ophiopogon japonicus* (L. f.), Ker-Gawl, root
RS: Regulating score
Rr: Ratio of recovery regulation
SHENMAI: Shenmai injection.
